# Type 2 Diabetes Mellitus Screening and Risk Factors Using Decision Tree: Results of Data Mining

**DOI:** 10.5539/gjhs.v7n5p304

**Published:** 2015-03-16

**Authors:** Shafi Habibi, Maryam Ahmadi, Somayeh Alizadeh

**Affiliations:** 1 Department of Health Information Management, School of Health Management and Information Sciences, Iran University of Medical Sciences, Tehran, Iran; 2 Health Management and Economics Research Center, School of Health Management and Information Sciences, Iran University of Medical Sciences, Tehran, Iran; 3 School of Industrial Engineering, Khajeh Nasir Toosi University of Technology, Tehran, Iran

**Keywords:** data mining, decision trees, Diabetes Mellitus Type 2, early diagnosis, risk factors

## Abstract

**Objectives:**

The aim of this study was to examine a predictive model using features related to the diabetes type 2 risk factors.

**Methods:**

The data were obtained from a database in a diabetes control system in Tabriz, Iran. The data included all people referred for diabetes screening between 2009 and 2011. The features considered as “Inputs” were: age, sex, systolic and diastolic blood pressure, family history of diabetes, and body mass index (BMI). Moreover, we used diagnosis as “Class”. We applied the “Decision Tree” technique and “J48” algorithm in the WEKA (3.6.10 version) software to develop the model.

**Results:**

After data preprocessing and preparation, we used 22,398 records for data mining. The model precision to identify patients was 0.717. The age factor was placed in the root node of the tree as a result of higher information gain. The ROC curve indicates the model function in identification of patients and those individuals who are healthy. The curve indicates high capability of the model, especially in identification of the healthy persons.

**Conclusions:**

We developed a model using the decision tree for screening T2DM which did not require laboratory tests for T2DM diagnosis.

## 1. Introduction

Type 2 diabetes is a chronic disease and one of the most common endocrine diseases including 90 to 95 percent of diabetic patients (American Diabetes Association, 2013) with different degrees of prevalence in various societies (King, Aubert, & Herman, 1998). It was recognized by an asymptomatic phase between the real onset of diabetic hyperglycemia and clinical diagnosis which lasts at least for 4-7 years (Brown, Critchley, Bogowicz, Mayige, & Unwin, 2012). Late or lack of diabetes diagnosis causes the increase of various chronic vascular complications (Heydari, Radi, Razmjou, & Amiri, 2010). Moreover, early diagnosis and prevention of diabetes reduces the high expenses associated with disease control and complication treatments and prevents hospital admissions due to its severe complications (Karter et al., 2003).

It is well known that about 30 to 80 percent of type 2 diabetic cases remain undiagnosed (Brown et al., 2012). Therefore, considering the prevention principle and in order to fight against the current widespread prevalence of diabetes, there is great emphasis on the significance of screening and recognition of those who might have diabetes or its higher probability without any symptoms. Timely diagnosis and prevention result in the decrease in mortality and prevention and decrease in the diabetes complications and improvement of quality of life (Gregg et al., 2001). The main challenge in diabetes screening, however, is the need to study and take blood samples of several people, which is expensive in terms of financial and personnel resources, and is beyond the capabilities of the health system, especially in the developing countries. Using data mining and knowledge discovery capabilities which identify latent patterns associated with diagnostic decisions from within the data could help in the prediction and diagnosis of the disease. In order to perform data mining and knowledge discovery, one needs large amounts of data related to the subject saved in the databases. Today, the importance of data saving and providing electronic records for the patients and those who refer to health centers is considered as a tool to improve the level of health of the people in society, and discussions about establishing and developing databases of Electronic Health Records (EHR) would pave the way for new studies in the health issues in society, even in the developing countries (DeVoe et al., 2011).

Data mining is a practical branch of artificial intelligence which discovers latent patterns through looking for relationships between features in large databases. The pattern discovered should be significant and provide advantages including economic ones (Witten & Frank, 2005). Classification is one of data mining methods used in the various studies in the health sector in order to build prediction models (Bellazzi, Ferrazzi, & Sacchi, 2011). The capability of these classification methods has been confirmed in discovering the relationships and patterns among the features in the databases and using the results and patterns thus discovered for diagnosis and prediction (Upadhyaya, Farahmand, & Baker-Demaray, 2013). In the screening of type 2 diabetes mellitus (T2DM), however, the capabilities of the classification techniques have not yet been demonstrated. The decision tree is one powerful and highly used method in classification which has found use in various medical arenas, in studies associated with the prediction and diagnosis and its application for prediction increased significantly (Liao, Chu, & Hsiao, 2012). As screening is performed at a wide level of society and requires great expense, facilities and time, further research in this field could help and improve health level of the society. Therefore, the aim of this study was to examine a predictive model using the features related to diabetes type 2 risk factors in order to help in the screening of diabetes using the decision tree.

## 2. Method

The data were obtained from the database of a web-based health center diabetes control system in Tabriz (center of East Azerbaijan province in Iran) designed for recording the data of those who had referred for diabetes screening from 2009 to 2011. According to the screening model communicated by the Iran Health Ministry to the health centers in the provinces and cities, those who had at least one risk factor of obesity or overweight, history of diabetes in first-degree relatives, hypertension, pregnancy, history of gestational diabetes, history of abortion, stillbirth, and birth of a child more than 4 kg, and past history of diabetes entered the system, and the tests required to diagnose diabetes were performed after recording the anthropometric data, blood pressure, and family history. Among 60,010 extracted records, 32,044 ones lacked value in the diagnosis field or each of the six fields used for entering the decision tree, which were all omitted. Furthermore, pregnant women (418 records) and those with a past history of diabetes (5,150 records) were excluded, as type 2 diabetes alone was considered in this study. After excluding the records mentioned above with the missed value, a total number of 22,398 records were used as diabetic, pre-diabetic and healthy categories, among which 924 persons (4.1%) had diabetes, 3,062 individuals (13.7%) were pre-diabetic, and 18,412 ones (82.2%) were healthy. The number of women and men were 15,019 and 7,379, respectively.

Features including age, sex, systolic and diastolic blood pressure, family history of diabetes, and body mass index (BMI) were used as inputs for the decision tree and the diagnosis feature were used as class. Unnecessary features such as glucose and triglyceride levels, etc. were omitted. The age variable was calculated and added using the date of birth and date of reference to the health center, and the same was performed for the BMI variables, using height and weight.

The technique of decision tree and J48 algorithm, which is the most important algorithm used for developing the decision tree in WEKA (3.6.10 version), was applied to develop the prediction model. J48 is Weka’s implementation of Quinlan’s C4.5 for building the decision tree (Witten & Frank, 2005). Knowledge flow environment is one of the WEKA environments for performing the algorithm mentioned. Due to the imbalance in the data class distribution, the boosting effective method (Witten & Frank, 2005) which is AdaboostM1 in Weka was used to identify the diabetic patients. The 10-fold Cross Validation method was used to validate the model, and Equations 1, 2 and 3 were used to calculate the Precision, Recall and Accuracy of the model, respectively.


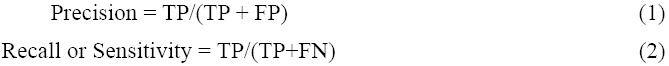


and


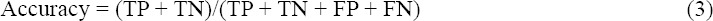


## 3. Results

After data preprocessing and preparation, a total of 22,398 records were used for data mining and developing the decision tree. The following results demonstrate the evaluation of the model developed in detail, according to which the precision of the model to identify patients was 0.717 and identifying those who were healthy was better and even more precise than identifying the patients. The Precision of patient identification was, however, just acceptable. The FP rate was low due to the higher identification of healthy individuals (Table 1).

**Table 1:** The results of the decision tree model evaluation

Evaluation measures	ROC Area	F-Measure	Recall	Precision	Accuracy	FP Rate
results	0.875	0.705	0.694	0.717	0.976	0.012

*Note.* ROC Area= Receiver Operating Characteristics Area, FP Rate= False Positives Rate.

The results indicated that the area below the ROC curve reached 0.875. The Kappa statistical value was about 0.69. According to the method mentioned, the confusion matrix is as follows:

As the confusion matrix reveals, the model could separate 98.8 percent (21,221) of healthy individuals from the other patients, while the number of patients identified was 69.4 percent (641 persons), which is less than that of the healthy ones. The whole total of those who belonged in the other class in this incorrect model was 2.4 percent (536 persons) (Table 2). According to the equations mentioned in the Methods Section, the precision and accuracy of the model was 71.7 and 97.6 percent, respectively.

**Table 2:** Confusion matrix of the decision tree model

Classes	Diabetic	Healthy
Healthy	253	21221
Diabetic	641	283

**Figure 1:**
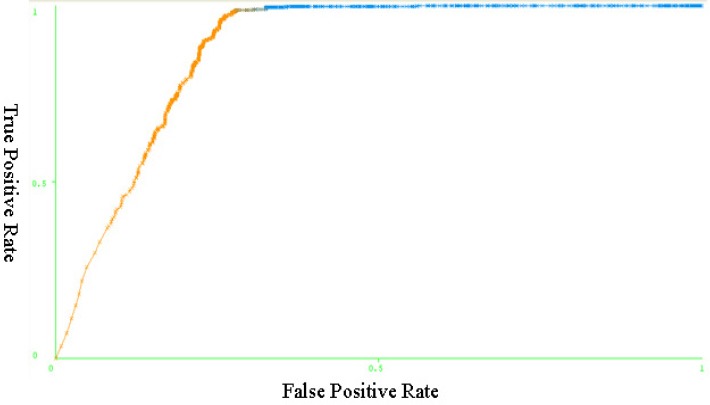
ROC curve-Performance of the J48

The ROC curve indicates the model function in identification of patients and those individuals who are healthy (Figure 1). The curve indicates high capability of the model, especially in identification of the healthy persons.

**Figure 2:**
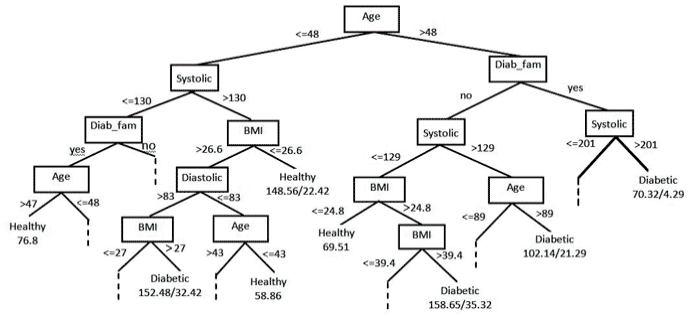
The resulting decision tree. Only the leaf nodes at the higher levels are displayed while the rest are indicated by dashes. The node “Family history of diabetes” was abbreviated to “Diab_fam”. The leaf nodes are assigned by one or two numbers where the former and latter indicate the number of correctly and incorrectly classified samples, respectively

The last iteration (10th) of the pruned tree included 289 leaves and the size of the tree was 577. Age feature was placed at the root node of the tree due to higher information gain. The tree was split into two branches: =<48 years old and >48 years old. Those with a family history of diabetes and systolic blood pressure were placed at the next level of the tree (Figure 2).

## 4. Discussion

T2DM is frequently undiagnosed until complications appear (American Diabetes Association, 2004). According to the Centers for Disease Control and Prevention (CDC), from among the 29.1 millions of US citizens who have diabetes, 8.1 million (27.8%) were undiagnosed in 2012 (Centers for Disease Control Prevention, 2014). Nearly one-third of the patients with T2DM who were newly diagnosed were observed to have complications with microvascular nephropathy, neuropathy and retinopathy (Raman et al., 2009). The results and models of studies similar to this project can facilitate early detection of T2DM and the prevention of potential complications associated with late diagnosis. The results of this study is consistent with findings of Meng et al., who used the features of risk factors such as eating habits, physical activity, and so on for T2DM prediction (Meng, Huang, Rao, Zhang, & Liu, 2013). However, most studies reported higher amounts of evaluation measures of accuracy, precision and sensitivity (Kahramanli & Allahverdi, 2008; Kayaer & Yıldırım, 2003; Lekkas & Mikhailov, 2010; K. Polat, Gnnes, & Arslan, 2008; Kemal Polat & Güneş, 2007; Prati, Batista, & Monard, 2009; Temurtas, Yumusak, & Temurtas, 2009). This was probably because of the features used in T2DM prediction. The features used in this study for T2DM prediction were indeed the same as those used in diabetes screening and recommended by most well-known authorities (American Diabetes Association, 2004; FRCSC, 2008; National Diabetes Information Clearinghouse (NDIC), 2014). Prior studies also have confirmed that these features are important predictor variables (Dong et al., 2011; Glumer et al., 2004; Li, Bergmann, Reimann, Bornstein, & Schwarz, 2009; Robinson, Agarwal, & Nerenberg, 2011). These studies, which aimed to identify and score the main variables affecting the development of diabetes, identified age and BMI as the main predictor variables and blood pressure measure, family history of DM and sex as the highest risk factor scores for the detection of undiagnosed diabetes (Brown et al., 2012).

The Pima Indians dataset has been used widely for data mining on diabetes mellitus. Out of the nine features two include plasma glucose and serum insulin. Findings from the past studies based on the Pima Indians or other datasets reported prediction models with high accuracy levels (Huang, McCullagh, Black, & Harper, 2007; Kahramanli & Allahverdi, 2008; Tama, Rodiyatul, & Hermansyah, 2013; Temurtas et al., 2009). This was due to the inclusion of plasma glucose and serum insulin. Fauci believes that the diagnosis of endocrine diseases is not yet established using the symptoms instead, and diagnosis is made by measuring the hormone levels secreted or their target (Fauci, 2008). The main diagnosis of diabetes mellitus is based on several techniques for measuring the plasma glucose or serum insulin level, confirming that the selection and application of the predictor features requires further attention.

Therefore, the prior researches, especially those which had employed those features related to the main diagnosis of diabetes, have compared the capability of the data mining techniques and algorithms and it was found that they were not to be considered in diabetes prediction or diagnosis. The current study tested the decision tree using real data and the features used in diabetes screening communicated by the Health Ministry of Iran to the health centers of the provinces and cities as defined diabetes risk factors so that the primary screening was performed by evaluating such features in those individuals who referred. The study demonstrated that the decision tree could be used in the screening and that it would help in patient screening by automating the screenings in the electronic systems.

## 5. Conclusion

We developed a model using the decision tree for the screening of T2DM that does not require laboratory tests for T2DM diagnosis. We used the J48 algorithm and the model proposed is different from the previous models for three reasons: 1) We used real dataset. 2) We used the features applied to primary screening, excluding those such as plasma glucose for the main diagnosis of T2DM. 3) Capability of the decision trees for T2DM screening. Although the exclusion of diabetes laboratory diagnostic tests features lowered the sensitivity and precision of the model proposed compared with models suggested in the literatures, this study is a step forward for the early diagnosis of diabetes without using diagnostic laboratory tests.
